# Leucine-Rich Repeat Kinase 2 (LRRK2) Stimulates IL-1β-Mediated Inflammatory Signaling through Phosphorylation of RCAN1

**DOI:** 10.3389/fncel.2017.00125

**Published:** 2017-05-11

**Authors:** Kyung A. Han, Lang Yoo, Jee Y. Sung, Sun A. Chung, Ji W. Um, Hyeyoung Kim, Wongi Seol, Kwang C. Chung

**Affiliations:** ^1^Department of Systems Biology, College of Life Science and Biotechnology, Yonsei UniversitySeoul, South Korea; ^2^Center for Pediatric Oncology, National Cancer CenterGoyang-si, South Korea; ^3^Department of Food and Nutrition, College of Human Ecology, Yonsei UniversitySeoul, South Korea; ^4^Department of Brain and Cognitive Sciences, Daegu Gyeongbuk Institute of Science and Technology (DGIST)Daegu, South Korea; ^5^InAm Neuroscience Research Center, Sanbon Medical Center, College of Medicine, Wonkwang UniversityGunpo-si, South Korea

**Keywords:** LRRK2, RCAN1, interleukin-1β, phosphorylation, NF-κB, Parkinson’s disease

## Abstract

Leucine-rich repeat kinase 2 (LRRK2) is a Ser/Thr kinase having mixed lineage kinase-like and GTPase domains, controlling neurite outgrowth and neuronal cell death. Evidence suggests that LRRK2 is involved in innate immune response signaling, but the underlying mechanism is yet unknown. A novel protein inhibitor of phosphatase 3B, RCAN1, is known to positively regulate inflammatory signaling through modulation of several intracellular targets of interleukins in immune cells. In the present study, we report that LRRK2 phosphorylates RCAN1 (RCAN1-1S) and is markedly up-regulated during interleukin-1β (IL-1β) treatment. During IL-1β treatment, LRRK2-mediated phosphorylation of RCAN1 promoted the formation of protein complexes, including that between Tollip and RCAN1. LRRK2 decreased binding between Tollip and IRAK1, which was accompanied by increased formation of the IRAK1-TRAF6 complex. TAK1 activity was significantly enhanced by LRRK2. Furthermore, LRRK2 enhanced transcriptional activity of NF-κB and cytokine IL-8 production. These findings suggest that LRRK2 might be important in positively modulating IL-1β-mediated signaling through selective phosphorylation of RCAN1.

## Introduction

Parkinson’s disease (PD) is the second-most common neurodegenerative disorder, and characterized by the formation of abnormal protein inclusions, referred as Lewy bodies (Moore et al., 2005). Numerous reports indicate that excessive inflammation in the brain is closely associated with the pathogenesis of and neural degeneration observed in PD. Many cytokines, including various interleukin (IL) subtypes and tumor necrosis factor α (TNF-α), are elevated in the brains and cerebrospinal fluid of PD patients (Mogi et al., 1994, 1996). During the progression of PD, neurotoxic molecules are released within neurons, which can trigger inflammatory signaling cascades.

Mutations in the gene encoding leucine-rich repeat kinase 2 (LRRK2) have also been linked to autosomal-dominant Parkinsonism (Li et al., 2014). Studies by others have demonstrated that LRRK2 is involved in the inflammatory response induced by α-synuclein or lipopolysaccharide (LPS). For example, rats deficient in LRRK2 are protected from dopaminergic neurodegeneration caused by α-synuclein over-expression or LPS exposure (Daher et al., 2014). Moreover, certain stimuli that induce inflammation are known to modulate the expression and activity of LRRK2. For example, interferon-γ and LPS treatment enhance LRRK2 expression and activity (Gardet et al., 2010; Moehle et al., 2012; Kuss et al., 2014). Recent studies also suggest that LRRK2 can regulate two key regulators of innate immune response, nuclear factor of activated T-cells (NFAT) and nuclear factor κB (NF-κB; Li et al., 2014).

Interestingly, TNF-α expression and the resultant cell death in the microglial cells from *LRRK2-R1441G* transgenic mouse were increased by LPS stimulation (Gillardon et al., 2012). On the contrary, the expression of interleukin-1β and cyclooxygenase-2 in microglial cells from *LRRK2*-knockout mouse was much reduced upon LPS treatment. The deletion of *LRRK2* also promoted the phosphorylation of NF-κB-inhibitory subunit p50 at S337 and the nuclear accumulation of NF-κB (Russo et al., 2015).

IL-1 receptors (IL-1Rs) and Toll-like receptors (TLRs) both commonly contain an intracellular Toll/IL-1R (TIR) domain and serve as major receptors of innate immunity and inflammation (Martin and Wesche, 2002). IL-1 signaling is initiated by the ligand-induced formation of a receptor complex consisting of IL-1R and the IL-1R accessory protein. In this process, MyD88 recruitment appears to constitute the first step in a series of protein-protein interactions at activated receptor complexes (Martin and Wesche, 2002). Under unstimulated conditions, Toll-interacting protein (Tollip) interacts with IL-1R-associated kinase 1 (IRAK1), inhibiting downstream TLR signaling (Martin and Wesche, 2002). During stimulation with IL-1, MyD88-IRAK1-Tollip complexes are recruited to the heterodimeric IL-1R. IRAK4 is simultaneously recruited to the receptor complex, phosphorylating IRAK1 and inducing IRAK1 auto-phosphorylation. Activated IRAK1 interacts with TNF receptor-associated factor 6 (TRAF6; Martin and Wesche, 2002). The TRAF6-IRAK1 complex dissociates from the receptor, and activated IRAK1 is subsequently degraded by the ubiquitin proteasome system (UPS). TRAF6 contains a RING domain and functions as an ubiquitin E3 ligase that conjugates Lys63-linked polyubiquitin chains to TRAF6 itself. Activated TRAF6 stimulates auto-phosphorylation of transforming growth factor-β-activated kinase 1 (TAK1), which then becomes fully activated. Regulatory kinases in downstream signaling pathways are phosphorylated by TAK1 (Martin and Wesche, 2002). Finally, NF-κB and numerous inflammatory cytokines themselves become activated (Lawrence, 2009).

Regulator of calcineurin 1 (RCAN1; also known as DSCR1) inhibits calcium-dependent protein phosphatase 3 (calcineurin), consequently affecting many cellular responses, including lymphocyte activation and neuronal and muscle development (Park et al., 2009). There are four transcripts of RCAN1, but the major transcriptional products are isoforms which include exon 1 (RCAN1-1) or 4 (RCAN1-4; Park et al., 2009). Although RCAN1-1 consists of 197 amino acid (RCAN1-1S), an additional start site has been found upstream of exon 1, which produces a protein with 252 amino acids (RCAN1-1L; Park et al., 2009). RCAN1 regulates the activities of several inflammatory transcription factors. For example, RCAN1 acts as a negative modulator of calcineurin, leading to inhibition of NFAT activity (Fuentes et al., 2000; Rothermel et al., 2000; Vega et al., 2002). RCAN1 also affects NF-κB activity and downstream cytokine signaling. For instance, RCAN1-1S interacts with Tollip, promoting its dissociation from the IRAK1 complex, which then stimulates NF-κB activity upon treatment with IL-1β (Lee et al., 2009).

Based on evidence suggesting a putative role of LRRK2 during inflammatory signaling, we investigated biochemical and functional interactions between LRRK2 and RCAN1 (specifically, RCAN1-1S), and a potential regulatory role for LRRK2 in RCAN1-mediated IL-1β inflammatory signaling. We determined RCAN1 to be a novel substrate of LRRK2, and that their interaction affects a key functional signalosome during IL-1β-mediated inflammatory signaling.

## Materials and Methods

### Materials

Peroxidase-conjugated anti-rabbit and anti-mouse antibodies were purchased from Millipore (Billerica, MA, USA). Dulbecco’s modified Eagle’s medium (DMEM), fetal bovine serum (FBS), fetal calf serum, and lipofectamine and PLUS reagents were purchased from Life Technologies (Grand Island, NY, USA). Anti-Myc, anti-GAPDH, anti-TAK1, anti-TRAF6 and anti-IRAK1 antibodies were purchased from Santa Cruz Biotechnology (Santa Cruz, CA, USA). Polyclonal anti-RCAN1 antibodies were purchased from ECM Biosciences (Versailles, KY, USA) and Santa Cruz Biotechnology. Anti-LRRK2, anti-phospho-LRRK2 (Ser935), and anti-phospho-TAK1 (Thr187) antibodies were purchased from Abcam (Cambridge, MA, USA) and Cell Signaling Technology (Beverly, MA, USA), respectively. Polyclonal and monoclonal anti-HA antibodies were purchased from Abnova (Tebu, France) and Covance (Powhatan, VA, USA), respectively. Protein A-Sepharose beads and glutathione sepharose 4B were purchased from GE Healthcare Life Science (Piscataway, NJ, USA). Enhanced chemiluminescence (ECL) reagent and [γ-32P] ATP were purchased from Perkin Elmer Life Sciences (Downers Grove, IL, USA). MG132 was purchased from A.G. Scientific (San Diego, CA, USA). Human recombinant IL-1β was purchased from Sigma (St. Louis, MO, USA).

### cDNA Constructs

Mammalian constructs encoding Myc-tagged wild-type LRRK2, its kinase-dead counterpart and two PD-associated pathogenic LRRK2 mutants (pcDNA3.1-Myc-LRRK2-WT, -D1994A, -G2019S and -R1441C, respectively) were generated, as described elsewhere (Shin et al., 2008; Heo et al., 2010). The mammalian expression vector for HA-tagged human wild-type RCAN1 (RCAN1-1S) was a kind gift from S. de la Luna (Genomics Regulation Center, Barcelona, Spain). Mammalian expression vectors encoding HA-tagged RCAN1 deletion mutants (HA-RCAN1^1–95^, HA-RCAN1^1–125^, HA-RCAN1^30–197^, and HA-RCAN1^90–197^) were constructed, as previously described (Lee et al., 2012). Bacterial expression vectors encoding GST-fused wild-type RCAN1 (pGEX4T-1-GST-RCAN1) and five deletion mutants (RCAN1^1–95^, RCAN1^1–125^, RCAN1^1–145^, RCAN1^1–160^ and RCAN1^1–175^) were produced by PCR amplification of wild-type RCAN1 and its deletion mutants using Prime STAR-HS DNA Polymerase (TAKARA, Shiga, Japan) and sub-cloning into the pGEX4T-1 vector. Bacterial constructs encoding GST-fused RCAN1 mutants having a substitution at Ser149, Thr151, Thr152, Thr154, or Ser162 with alanine, respectively, were generated through PCR amplification of pGEX4T-1-GST-RCAN1-WT as a template. Site-directed mutagenesis reactions were performed using the Prime^®^STAR HS DNA polymerase (Takara Bio Inc., Shiga, Japan).

### Cell Culture and DNA Transfection

LRRK2 +/+ and LRRK2 −/− mouse embryonic fibroblasts (MEFs) were kindly provided by D.R. Alessi (University of Dundee, Dundee, Scotland), and human embryonic kidney 293 (HEK293) cells stably expressing type 1 IL-1R (HEK293/IL-1RI) were a generous gift from G. Takaesu (Keio University, Tokyo, Japan). RAW264.7 cells, HEK293 cells, and LRRK2 +/+ and −/− MEFs were cultured in DMEM containing 10% FBS, 100 unit/ml penicillin, and 100 μg/ml streptomycin at 37°C in 5% CO_2_. HEK293/IL-1RI cells were also cultured in DMEM containing 10% fetal calf serum, 100 unit/ml penicillin, and 100 μg/ml streptomycin at 37°C in 5% CO_2_. All DNA transfections were performed using the Lipofectamine and PLUS reagent according to the manufacturer’s protocol.

### Immunoprecipitation and Western Blot Analysis

Cells were rinsed with ice-cold phosphate-buffered saline (PBS), harvested in 1% Nonidet P40 lysis buffer (50 mM Tris, pH 7.5, 1% Nonidet P40, 150 mM NaCl, 10% glycerol and protease inhibitor cocktail including 1 mM Na_3_VO_4_, 1 μg/ml leupeptin, 1 μg/ml aprotinin, 10 mM NaF and 0.2 mM phenylmethylsulfonyl fluoride), and briefly sonicated. Brain lysates were obtained from P28 mouse and brain tissues were lysed by RIPA buffer (50 mM Tris (pH 7.4), 150 mM NaCl, 1% Triton X-100, 0.5% sodium deoxycholate, 0.1% SDS, and protease inhibitor cocktail). All experimental protocols using mice were approved by the Institutional Animal Care and Use Committee of Yonsei University (the approval reference number: 2007–0004). Lysates were collected after centrifugation for 20 min at 4°C. For immunoprecipitation, 1 μg of the appropriate antibody was incubated overnight at 4°C with 0.8 mg of cell extracts prepared in cell lysis buffer. The samples were incubated with 30 μl of a 1:1 suspension of protein A-Sepharose beads for 2 h at 4°C with gentle rotation. Beads were pelleted by centrifugation at 10,000× *g* for 30 s at 4°C, and washed three times with 1% Nonidet P40 lysis buffer. Immunocomplexes were dissociated by boiling in 2× SDS-PAGE sample buffer, separated by SDS-PAGE, and transferred to a nitrocellulose membrane. Membranes were then blocked in TBST buffer (20 mM Tris, pH 7.5, 137 mM NaCl, and 0.1% Tween 20) containing 5% nonfat dry milk for 1 h at room temperature, and incubated overnight at 4°C in TBST buffer containing 3% nonfat dry milk and the appropriate primary antibody. Membranes were washed three times in TBST and incubated with the secondary IgG-coupled horseradish peroxidase antibody for 2 h at room temperature. Membranes were washed three times with TBST and the signal visualized using ECL reagent. Band intensities were measured using Multi Gauge v3.1 software (Fujifilm Life Science, Tokyo, Japan).

### Phos-Tag Immunoblotting

After DNA transfection, cells were rinsed in ice-cold PBS, and then lysed with the lysis buffer containing 0.2% Nonidet P40, 50 mM Tris (pH 7.4), 150 mM NaCl, 10% glycerol and protease inhibitor cocktail. Samples containing phosphorylated RCAN1 were separated by 10% SDS-PAGE containing 25 mM Phos-tag (Wako Pure Chemical Industries, Osaka, Japan). Phos-tag immunoblotting was performed according to the manufacturer’s protocol.

### GST Pull-Down Assay

GST pull-down assays were performed by incubating GST or GST-fused RCAN1 immobilized onto GST beads for 2 h at 4°C with the cell lysates. The mixtures were washed three times with wash buffer (25 mM Tris-HCl, pH 7.5, 1 mM dithiothreitol, 30 mM MgCl_2_, 40 mM NaCl, and 1% Nonidet P40). Bound proteins were eluted with 2× SDS buffer, separated by SDS/PAGE, and then subjected to Western blot analysis using an anti-Myc antibody.

### RNA Interference

Small interference RNAs (siRNA) targeting human RCAN1 (siRNA no. 1044226) and scrambled siRNA (catalog no. SN-1013) were purchased from Bioneer (Daejeon, South Korea). HEK293/IL-1RI cells were transfected with 200 nM siRNA using lipofectamine RNAiMAX (Life Technologies) according to the manufacturer’s protocol.

### Luciferase Reporter Assay

HEK293/IL-1RI and LRRK2 +/+ and LRRK2 −/− MEF cells were mock-transfected or transfected with Myc-LRRK2 wild-type or kinase-dead constructs. NF-κB-dependent firefly luciferase reporter and effector plasmids were co-transfected into the cells along with the Renilla luciferase plasmid. After 24 h, cells were treated with IL-1β for 6 h and then harvested in a passive lysis buffer. Luciferase assays were performed using the Dual-Luciferase Reporter Assay System (Promega, Madison, WI, USA). Relative luciferase activity was calculated by dividing firefly luciferase activity by Renilla luciferase activity. Data represented three independent experiments performed in triplicate.

### *In Vitro* Kinase Assay

HEK293 cells were transfected for 24 h with plasmids encoding Myc-tagged wild-type LRRK2 or its kinase-dead mutant (LRRK2-D1994A). Cells were lysed in 1% Nonidet P40 lysis buffer and cell lysates were immunoprecipitated overnight at 4°C with an anti-Myc antibody. The immunocomplexes were incubated with 30 μl of a 1:1 protein A-Sepharose bead suspension for 2 h at 4°C with gentle inversion. Beads were centrifuged and washed twice with lysis buffer, and washed twice with 1× LRRK2 kinase reaction buffer (20 mM HEPES, pH 7.4, 15 mM MgCl_2_, 5 mM EGTA, 0.1% Triton X-100, 0.5 mM DTT, and 1 mM glycerophosphate). The samples were then mixed with 2 μg of bacterial recombinant GST-fused wild-type RCAN1 or its deletion mutants and 1× LRRK2 kinase reaction buffer containing 10 μM cold-ATP. The *in vitro* kinase reaction was initiated by the addition of 5 μCi (γ-32P) ATP. The reaction was allowed to proceed for 15 min at 30°C before being terminated by the addition of SDS-PAGE sample buffer. Protein samples were resolved by SDS-PAGE and incorporated (γ-32P) radioisotope was detected by autoradiography.

### Real-Time PCR Analysis for IL-8 mRNA Expression

Total RNAs were converted into cDNAs using Primescript RT master Mix kit (TAKARA). cDNA was incubated with SYBR Green Real-time PCR master mix (Toyobo Co. Ltd., Osaka, Japan) containing 10 pg/ml of the forward and reverse primers, and then amplified using the Light Cycler PCR system (Roche Applied Sciences, Indianapolis, IN, USA). Sequences of the IL-8 primers used were 5′-ATGACTTCCAAGCTGGCCGTGGCT-3′ (forward) and 5′-TCTCAGCCCTCTTCAAAAACTTCT-3′ (reverse), generating a 297-base pair PCR product. GAPDH was used as an internal control, the forward primer being 5′-GAAGGTGAAGGTCGGAGT-3′ and the reverse primer being 5′-GAAGATGGTGATGGGATTC-3′, generating a 226-base pair PCR product.

### Enzyme-Linked Immunosorbent Assay

After DNA transfection, the amount of secreted IL-8 was determined using an ELISA kit (Biosource, Camarillo, CA, USA), according to the manufacturer’s protocol.

### Statistical Analysis

Data are represented as means ± SEM. Group means were compared using Student’s *t*-tests. *P* values < 0.05 were considered statistically significant.

## Results

### LRRK2 Physically Interacts with RCAN1-1S

Both LRRK2 and RCAN1-1S regulate NF-κB transcriptional activity in the IL-1R/TLR signaling pathway (Lee et al., 2009; Li et al., 2014). Therefore, we sought to determine if there were any biochemical interactions between LRRK2 and RCAN1. We also investigated the potential functional and physiological significance, if any, of such an interaction, with particular focus on regulation of the IL-1β-mediated inflammatory signaling pathway.

**Figure 1:**
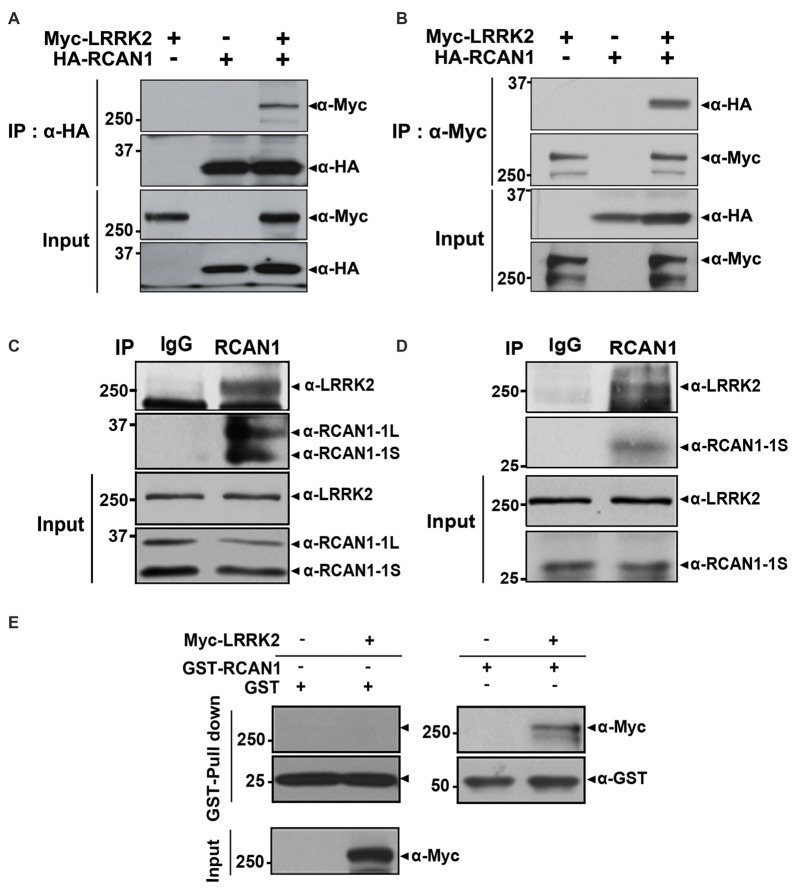
**Leucine-rich repeat kinase 2 (LRRK2) interacts with RCAN1. (A,B)** Human embryonic kidney 293 (HEK293) cells were transfected for 24 h with plasmid encoding Myc-tagged wild-type LRRK2 and/or HA-tagged RCAN1. Total cell lysates were immunoprecipitated with anti-HA **(A)** or anti-Myc **(B)** antibodies, and immunocomplexes were analyzed using anti-Myc **(A)** or anti-HA **(B)** antibodies. Expression of proteins were determined using Western blotting with anti-Myc or anti-HA IgGs. **(C)** HEK293/IL-1RI cell lysates were immunoprecipitated with an anti-RCAN1 antibody, followed by immunoblotting with an anti-LRRK2 antibody. As a negative control, cell lysates were immunoprecipitated with pre-immune IgG (IgG), as indicated. Expression of LRRK2 and RCAN1 (RCAN1-1S and RCAN1-1L) in cell extracts was determined using immunoblotting with anti-LRRK2 or anti-RCAN1 antibodies. **(D)** Immunoprecipitation of mouse brain lysates was performed with an anti-RCAN1 antibody, followed by immunoblotting with an anti-LRRK2 antibody. As a control, cell lysates were immunoprecipitated with pre-immune IgG (IgG). Expression of LRRK2 and RCAN1-1S in tissue lysates was determined using immunoblotting with anti-LRRK2 or anti-RCAN1 antibodies. **(E)** GST pull-down assays were performed by incubating cell lysates prepared after DNA transfection with Myc-LRRK2-WT or empty vector for 24 h with GST-RCAN1-immobilized glutathione-sepharose. Bound complexes were analyzed using Western blotting with an anti-Myc antibody; GST served as a negative control. Purification of GST-fused RCAN1 and the presence of Myc-LRRK2 in cell extracts were confirmed using Western blotting with anti-GST or anti-Myc antibodies, respectively.

We first determined whether LRRK2 interacts with RCAN1 in mammalian cells using co-immunoprecipitation (co-IP) assays. After transfection of HEK293 cells with Myc-tagged LRRK2 and/or HA-tagged RCAN1 for 24 h, cell lysates were immunoprecipitated using anti-HA or anti-Myc antibodies. Immunoblot analyses found that ectopically expressed LRRK2 binds to RCAN1 in HEK293 cells (Figures 1A,B). Co-immunoprecipitation analyses also demonstrated that endogenous LRRK2 binds to endogenous RCAN1, whereas there was no obvious interaction in immunocomplexes from samples prepared with IgG as a negative control (Figure 1C). We determined that LRRK2 interacts with RCAN1 in the mouse brain (Figure 1D). Furthermore, *in vitro* GST pull-down assays performed on HEK293 cell lysates with GST-fused RCAN1 demonstrated that LRRK2 directly binds RCAN1, whereas, this interaction was not observed in samples prepared with GST as a control (Figure 1E). Collectively, these data suggest that LRRK2 specifically interacts with RCAN1 in mammalian cells.

**Figure 2:**
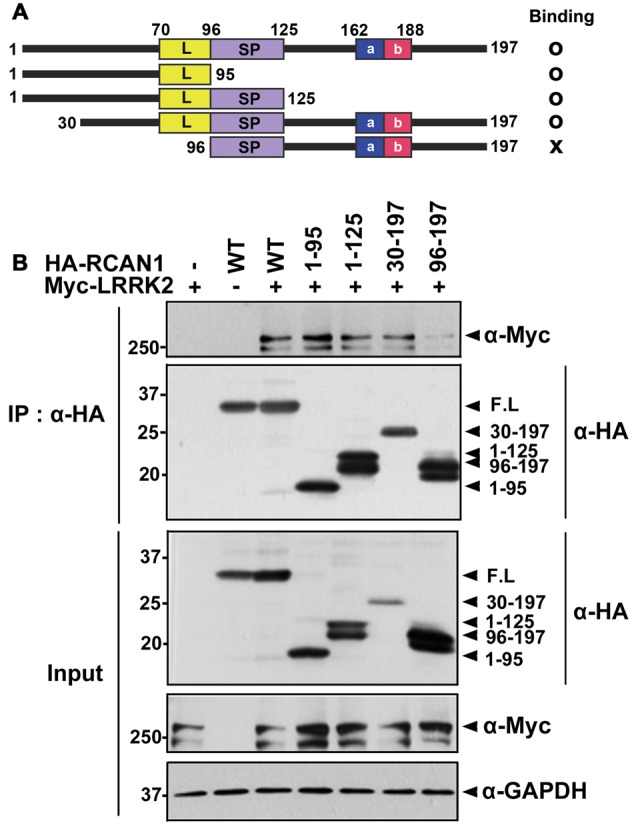
**The N-terminal 30–95 region of RCAN1 is important for binding with LRRK2. (A)** Diagram of HA-tagged wild-type RCAN1-1S, its deletion mutants, and a summary of the binding assay results. RCAN1-1S consists of an N-terminal amphipathic leucine repeat (L) domain, a central span of 31 amino acids containing a serine-proline (SP) repeat, a C-terminal acidic region (a), and a cluster of basic amino acids (b). **(B)** HEK293 cells were transfected for 24 h with Myc-LRRK2 alone or in combination with various HA-tagged deletion RCAN1 mutants, as indicated. Cell lysates were immunoprecipitated using anti-HA antibody. Immunocomplexes were analyzed using Western blotting with anti-Myc antibody, as indicated. Expression of Myc-LRRK2 and HA-RCAN1 was determined using Western blotting with anti-Myc or anti-HA antibodies. GAPDH served as a loading control.

To determine which domain(s) of RCAN1 interact with LRRK2, deletion mutants of RCAN1 (RCAN1^1–95^, RCAN1^1–125^, RCAN1^30–197^, and RCAN1^96–197^) were generated and their ability to interact with LRRK2 was tested (Figure 2A). Co-IP assays found that wild-type LRRK2 binds several RCAN1 peptides, specifically, RCAN1^1–95^, RCAN1^1–125^, and RCAN1^30–197^, as well as the full-length RCAN1 (Figures 2A,B). However, RCAN1^96–197^ exhibited greatly diminished interaction with LRRK2 (Figures 2A,B). These results suggest that the N-terminal 30–95 amino acid region of RCAN1 is necessary for LRRK2 binding.

### LRRK2 Directly Phosphorylates RCAN1

**Figure 3:**
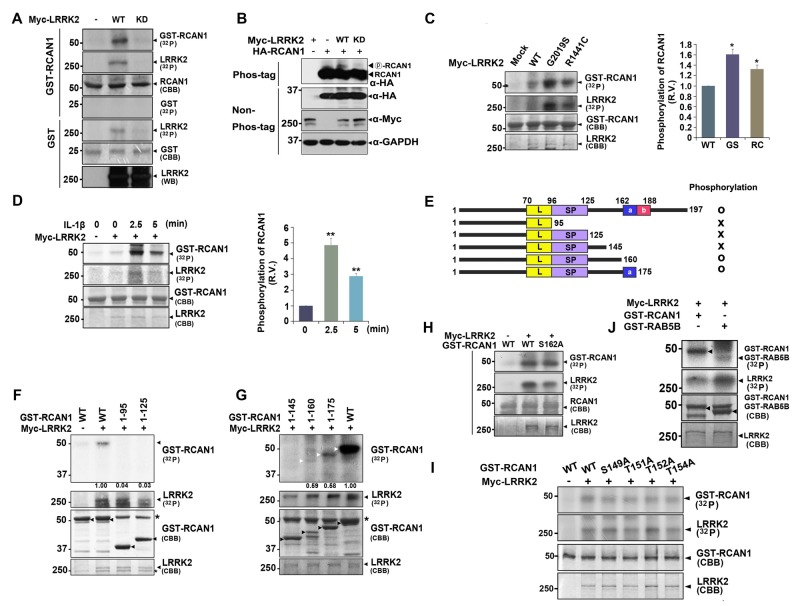
**LRRK2 directly phosphorylates RCAN1, which is enhanced by IL-1β treatment. (A)** HEK293 cells were mock-transfected or transfected for 24 h with Myc-tagged wild-type LRRK2 (LRRK2-WT) or its kinase-dead mutant having the point mutation of D1994A (LRRK2-KD). Cell lysates were immunoprecipitated with an anti-Myc antibody, and *in vitro* kinase assay was performed. **(B)** HEK293 cells were transfected for 24 h with HA-RCAN1, Myc-LRRK2-WT, or Myc-LRRK2-KD alone or in combination. Cell lysates were separated on a phos-tag gel, followed by western blotting with an anti-HA antibody (phos-tag). Expression of proteins in cell lysates was identified using western blotting with anti-Myc or anti-HA antibodies (non-phos-tag). GAPDH served as a loading control. **(C)** HEK293 cells were transfected for 24 h with plasmids encoding Myc-tagged LRRK2-WT, or the LRRK2-G2019S or LRRK2-R1441C mutants, and *in vitro* kinase assay was performed. The intensity of phosphorylated RCAN1 bands was quantified by densitometry using Multi Gauge v3.1 software (right panel in **C,D**). Individual band intensities were normalized to that for total RCAN1 for each experimental group. Error bars indicate ± SEM in triplicate experiments (*p < 0.05; **p < 0.01). **(D)** HEK293/IL-1RI cells were mock-transfected or transfected for 24 h with Myc-LRRK2-WT, and treated with 10 ng/ml IL-1β for the indicated times. Cell lysates were immunoprecipitated with an anti-Myc antibody, and *in vitro* kinase assay was performed. **(E)** Schematic representation of wild-type RCAN1, its deletion mutants, and a summary of the phosphorylation assay results. **(F,G)** After HEK293 cells were mock-transfected or transfected for 24 h with Myc-LRRK2-WT, *in vitro* kinase assay was performed with GST-fused RCAN1 deletion mutants, as indicated. Asterisks indicate non-specific proteins. **(H–J)** HEK293 cells were mock-transfected or transfected for 24 h with Myc-LRRK2-WT, and *in vitro* kinase assay was performed with recombinant GST-RCAN1-WT and GST-RCAN1-S162A **(H)**, GST-RCAN1-WT, GST-RCAN1-S149A, RCAN1-T151A, RCAN1-T152A, and RCAN1-T154A **(I)**, or GST-RAB5B **(J)** as a substrate.

The finding that LRRK2 interacts with RCAN1 raised the possibility that LRRK2, a protein kinase, can phosphorylate RCAN1. To test this hypothesis, *in vitro* kinase assays were performed using lysates from HEK293 cells transfected with Myc-tagged wild-type LRRK2 or a kinase-dead mutant (LRRK2-D1994A or LRRK2-KD) and bacterial recombinant GST-RCAN1 as a substrate. After incubation with anti-Myc immunocomplexes, GST-RCAN1, and (γ-^32^P) ATP, autoradiography results showed that wild-type LRRK2, but not the LRRK2-KD mutant, directly phosphorylates RCAN1 *in vitro* (Figure 3A).

We further tested whether LRRK2 phosphorylates RCAN1 in transformed cell lines. HEK293 cells were transfected with HA-RCAN1 alone or with either Myc-tagged wild-type LRRK2 or Myc-LRRK2-KD. Analyses of cell lysates using phos-tag gel, which separates non-phosphorylated proteins from phosphorylated ones, depending on differential motility rates, showed that levels of phosphorylated RCAN1 were considerably higher in the presence of wild-type LRRK2, but not LRRK2-KD (Figure 3B). Two of the most common pathogenic PD-associated LRRK2 mutants, LRRK2-G2019S and LRRK2-R1441C, have been shown to have enhanced kinase activity. Both LRRK2-G2019S and LRRK2-R1441C increased RCAN1 phosphorylation by ~1.6 and ~1.3-fold, respectively (Figure 3C).

Previous reports indicate that RCAN1 and LRRK2 are closely related to IL-1R/TLR signaling (Lee et al., 2009; Li et al., 2014). Therefore, we next addressed whether treatment with IL-1β affects or accelerates RCAN1 phosphorylation by LRRK2. To test this hypothesis, HEK293/IL-1RI cells stably expressing the type I IL-1β receptor, which exhibits a robust response to IL-1β stimulation (Cao et al., 1996), were utilized. After transfection with Myc-tagged LRRK2 for 24 h, HEK293/IL-1RI cells were treated with 10 ng/ml IL-1β and anti-Myc immunocomplexes were isolated. *In vitro* kinase assays followed by autoradiography showed that stimulation with IL-1β promoted LRRK2 auto-phosphorylation and LRRK2-mediated RCAN1 phosphorylation (Figure 3D). These results indicate that treatment with IL-1β stimulates RCAN1 phosphorylation by LRRK2 through an IL-1R-mediated signaling pathway.

To identify which region(s) in RCAN1 is important for LRRK2-mediated phosphorylation, *in vitro* kinase assays were performed using truncated GST-fused RCAN1 mutants. HEK293 cells were mock-transfected or transfected with Myc-tagged LRRK2, and cell lysates were immunoprecipitated with an anti-Myc antibody. *In vitro* kinase assays using anti-Myc-LRRK2 immunocomplexes and recombinant GST-fused RCAN1 deletion mutants demonstrated that RCAN1^1–95^, RCAN1^1–125^ and RCAN1^1–145^ were not phosphorylated by LRRK2 (Figures 3E–G), whereas RCAN1^1–175^ and RCAN1^1–160^ are phosphorylated weakly or much less, respectively (Figure 3G). These results indicated that the C-terminal 145–160 or 145–175 amino acid region of RCAN1 contains target(s) for LRRK2-mediated phosphorylation. As the 160–175 region contains only one Ser/Thr residue (Ser-162), we further examined whether it is directly phosphorylated by LRRK2. *In vitro* kinase assays by using the GST-fused RCAN1 mutant having a substitution at Ser162 with alanine (GST-RCAN1-S162A mutant) as a substrate revealed that this fragment is still phosphorylated by LRRK2 (Figure 3H). As the 145–160 region contains four additional Ser/Thr residues for the phosphorylation (i.e., Ser149, Thr151, Thr152 and Thr154), we then generated four RCAN1 mutants having a substitution of each site with alanine, respectively. When we assessed the effect of LRRK2-mediated phosphorylation on these mutations, unexpectedly, all these point-mutants were still and substantially phosphorylated by LRRK2 (Figure 3I). These results indicate that LRRK2-targeted residue is not simply limited to the region spanning amino acids 145–175. These results also suggest that LRRK2-mediated phosphorylation of deletion mutants failed to reflect the phosphorylation pattern of wild-type RCAN1.

Next, we examined the phosphorylation efficiency of LRRK2 toward RCAN1 through comparison with RAB5B, another well-known target of LRRK2 (Yun et al., 2015). As shown in Figure 3J, *in vitro* kinase assays demonstrated that LRRK2 phosphorylated GST-RCAN1 more efficiently than RAB5B, confirming that RCAN1 is a novel substrate of LRRK2 kinase.

### LRRK2 Increases Association between RCAN1-1S and Tollip

A previous report has found that RCAN1-1S interacts with Tollip and promotes IL-1β-mediated inflammatory signaling (Lee et al., 2009). Consistent with this finding, our present results show that IL-1β treatment potentiates LRRK2-mediated phosphorylation of RCAN1. Taken together, these observations suggest that LRRK2 can modulate IL-1R-mediated downstream signaling.

**Figure 4:**
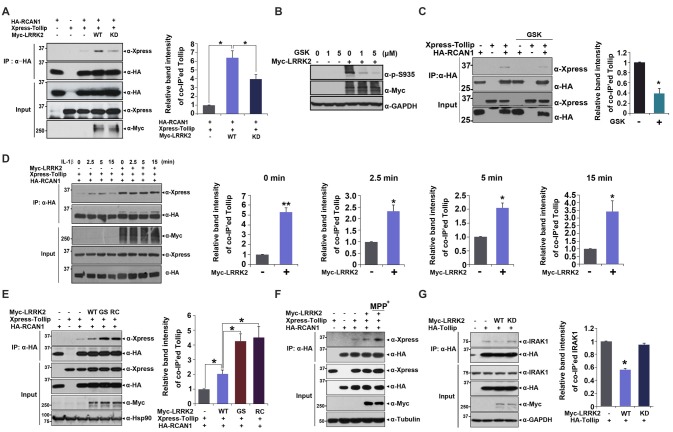
**LRRK2 increases interaction between Tollip and RCAN1. (A)** After HEK293 cells were transfected for 24 h with HA-RCAN1, Xpress-Tollip, or Myc-LRRK2 alone or in combination, co-IP experiment was performed, as indicated. Quantitation of co-IP’ed Tollip band intensities was presented (right panel in **A** and **C–E**; Error bars indicate ± SEM in triplicate experiments *p < 0.05; **p < 0.01). **(B)** HEK293 cells were mock-transfected or transfected for 24 h with Myc-LRRK2, and treated for 5 h with the indicated concentrations of GSK2578215A (GSK). Cells were then lysed and LRRK2 activities were determined by immunoblotting of cell lysates with phosphor-S935 LRRK2 antibody. **(C)** HEK293 cells were transfected for 24 h with HA-RCAN1 or/and Xpress-Tollip, and treated for 5 h with 1 μM GSK2578215A. Co-IP experiment was then performed, as indicated. **(D)** HEK293/IL-1RI cells were transfected for 24 h with HA-RCAN1, Xpress-Tollip, or Myc-LRRK2 alone or in combination, and treated with 10 ng/ml IL-1β for the indicated times. Immunoprecipitation of cell lysates was performed with an anti-HA antibody, followed by immunoblotting with an anti-Xpress antibody. Co-IP experiment was then performed, as indicated. **(E)** HEK293 cells were transfected for 24 h with HA-RCAN1 and Xpress-Tollip alone or in combination with either Myc-tagged wild-type LRRK2 (LRRK2-WT) or LRRK2-G2019S or LRRK2-R1441C mutants. Co-IP experiment was then performed, as indicated. **(F)** SH-SY5Y cells were transfected for 48 h with HA-RCAN1, Xpress-Tollip, or Myc-LRRK2 alone or in combination, and treated for 10 h with 500 μM MPP^+^. **(G)** HEK293 cells were transfected for 24 h with HA-Tollip alone or with Myc-LRRK2-WT or Myc-LRRK2-KD. Total cell lysates were immunoprecipitated with an anti-HA antibody, and immunocomplexes were probed with an anti-IRAK1 antibody. The intensities of co-IP’ed IRAK1 bands (right panel) were quantified by densitometry using Multi Gauge v3.1 software. Error bars indicate ± SEM in triplicate experiments (*p < 0.05).

To investigate a putative regulatory role for LRRK2 in inflammatory response, we assessed whether LRRK2 modulates interaction between RCAN1 and Tollip in response to IL-1β treatment. HEK293/IL-1RI cells were transfected with HA-RCAN1 and Xpress-Tollip alone or with LRRK2-WT or LRRK2-KD. Co-immunoprecipitation assays showed that there was increased interaction between RCAN1 and Tollip in the presence of LRRK2-WT, compared with control cells. However, RCAN1 binding to Tollip was reduced by LRRK2-KD (Figure 4A). To further verify the role of native LRRK2 in RCAN1-Tollip interaction, we tested the effect of GSK2578215A (GSK), a potent and highly selective inhibitor of LRRK2 kinase, on their binding. As a control, pretreatment of cells with GSK at 1 μM for 5 h sufficiently inhibits the LRRK2 activity, as measured by LRRK2 phosphorylation at Ser935 (Deng et al., 2011; Figure 4B). As shown in Figure 4C, GSK treatment attenuated the interaction between RCAN1 and Tollip. In addition, the increased RCAN1 binding to Tollip in the presence of LRRK2 was much enhanced and sustained longer after IL-1β treatment (Figure 4D).

We then investigated whether two pathogenic LRRK2 mutations affect association between RCAN1 and Tollip. We evaluated binding affinity between RCAN1 and Tollip in response to treatment with wild-type LRRK2 and two PD-associated mutants. Co-IP analyses showed that the mutants LRRK2-G2019S and LRRK2-R1441C increased formation of RCAN1-Tollip complexes, much more than did wild-type LRRK2 (Figure 4E). These data are consistent with findings regarding LRRK2-mediated RCAN1 phosphorylation. They also suggest that the binding of RCAN1 to Tollip is dependent upon the kinase activity of LRRK2.

To determine whether the interaction between RCAN1 and Tollip is associated with the progression of PD, we tested the effect of neurotoxin, MPP^+^, which causes symptoms of PD by destroying dopaminergic neurons in the substantia nigra of the brain, on LRRK2-induced RCAN1-Tollip interaction in neuroblastoma SH-SY5Y cell line. Interestingly, MPP^+^ treatment further increased the binding affinity between RCAN1 and Tollip which is stimulated by LRRK2 (Figure 4F). These results suggest that the formation of RCAN1-Tollip complex which is affected by LRRK2 might play a role in the pathogenesis of PD.

In addition to modulation of IL-1R-binding complex formation, Tollip also binds to IRAK1 and acts as a negative modulator of downstream IRAK1 signaling. Thus, we further examined whether LRRK2 can regulate the formation of Tollip-IRAK1 complexes. HEK293/IL-1RI cells were transfected with HA-Tollip alone or with Myc-tagged LRRK2-WT or the LRRK2-KD mutant; cell lysates were immunoprecipitated using an anti-HA antibody. Immunoblot analyses of anti-HA immunocomplexes using an anti-IRAK1 antibody showed that LRRK2 promoted the dissociation of Tollip-IRAK1 complexes (Figure 4G). These results suggest that LRRK2 positively regulates association between RCAN1 and Tollip, while inhibiting association between Tollip and IRAK1, which consequently potentiates IL-1R-mediated downstream signaling.

### LRRK2 Promotes the Assembly of IRAK1-TRAF6

Stimulation of IL-1R/TLR with IL-1β results in activation of IRAK1 and subsequent recruitment of TRAF6 to IRAK1 complexes (Jiang et al., 2002). The IRAK1-TRAF6 complex dissociates from the activated heterodimer receptor. Activated IRAK1 is degraded by the UPS through the Skp1-Cullin1-F-box-β-TrCP complex, while TRAF6 activates downstream inflammatory signaling (Cui et al., 2012). Previous reports also indicate that K63-linked ubiquitination of IRAK1 occurs during stimulation with IL-1, and that it is important for activation of NF-κB and the IκB kinase (Conze et al., 2008; Windheim et al., 2008).

**Figure 5:**
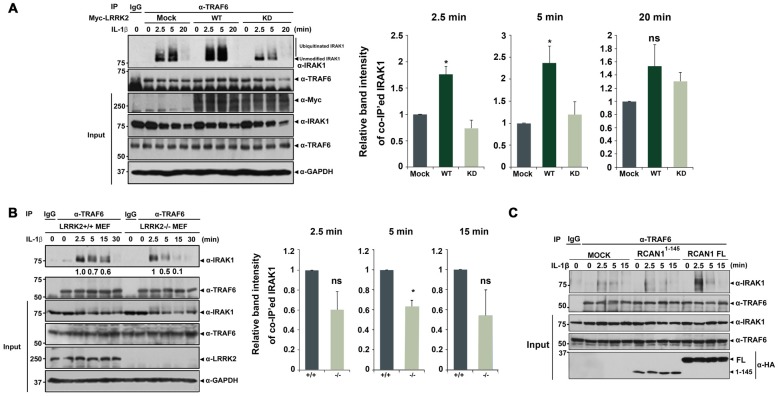
**LRRK2 facilitates assembly of the IRAK1-TRAF6 complex. (A)** HEK293/IL-1RI cells were either mock-transfected or transfected for 24 h with Myc-tagged LRRK2-WT or the LRRK2-KD mutant and treated with 10 ng/ml IL-1β for the indicated times. Cell lysates were immunoprecipitated with an anti-TRAF6 antibody and immunoblotted with an anti-IRAK1 antibody. Expression of Myc-LRRK2-WT, Myc-LRRK2-KD, endogenous TRAF6, and IRAK1 in cell lysates was determined using immunoblotting with anti-Myc, anti-TRAF6, or anti-IRAK1 antibodies (left panel). **(B)** *LRRK2* +/+ or *LRRK2* −/− Mouse embryonic fibroblasts (MEFs) were treated with 50 ng/ml IL-1β for the indicated times. Cell lysates were immunoprecipitated with an anti-TRAF6 antibody and immunoblotted with an anti-IRAK1 antibody. As a control, cell lysates were immunoprecipitated with pre-immune IgG (IgG), as indicated. Endogenous LRRK2, TRAF6 and IRAK1 levels were determined using immunoblotting with their respective antibodies (left panel). The intensity of co-IP’ed IRAK1 bands was quantified by densitometry using Multi Gauge v3.1 software (right panel in **A,B**; *n* = 3). Individual band intensities were normalized to that for the co-IP’ed TRAF6 for each experimental group. Error bars indicate ± SEM in triplicate experiments (*p < 0.05). **(C)** HEK293/IL-1RI cells were either mock-transfected or transfected for 24 h with HA-tagged full-length RCAN1 (RCAN1-FL) or RCAN1^1–145^ mutant, and treated with 10 ng/ml IL-1β for the indicated times. Cell lysates were immunoprecipitated with anti-TRAF6 antibody and immunoblotted with anti-IRAK1 antibody. Expression of HA-RCAN1-FL, HA-RCAN1^1–145^, endogenous TRAF6, or IRAK1 in cell lysates was determined using immunoblotting with anti-HA, anti-TRAF6, or anti-IRAK1 antibodies.

Based on these observations, we next investigated whether LRRK2 also modulates the assembly of IRAK1-TRAF6 complexes. HEK293/IL-1RI cells were mock-transfected or transfected with Myc-tagged LRRK2-WT or Myc-LRRK2-KD mutant for 24 h. Cells were then treated for 6 h with the proteasome inhibitor MG132 to prevent IRAK1 degradation and for 2.5, 5, or 20 min with IL-1β. Immunoprecipitation of cell lysates was performed using an anti-TRAF6 antibody, followed by immunoblotting with anti-IRAK1 antibodies. LRRK2-WT enhanced the association of IRAK1 and TRAF6 at 2.5 and 5 min after IL-1β treatment; the LRRK2-KD mutant had no significant effect on this interaction (Figure 5A). Consistent with this finding, when cells were treated with MG132, we also observed formation of smeared IRAK1 bands, indicating K48- and K63-linked polyubiquitination of IRAK1 in response to IL-1β stimulation (Figure 5A).

We next examined whether *LRRK2* deletion inversely affects interaction between endogenous IRAK1 and endogenous TRAF6. After treatment of *LRRK2* +/+ and *LRRK2* −/− MEFs with IL-1β, we evaluated IRAK1-TRAF6 complex formation. Co-IP analyses demonstrated that levels of endogenous IRAK1-TRAF6 complexes were significantly reduced in *LRRK2* −/− MEF after stimulation with IL-1β, compared with *LRRK2* +/+ MEFs (Figure 5B). These data indicate that LRRK2 facilitates formation of IRAK1-TRAF6 complexes in response to IL-1β stimulation.

To further investigate the specific effects of LRRK2 on RCAN1 function in response to IL-1β stimulation, we utilized RCAN1^1–145^ deletion mutant, which is not phosphorylated by LRRK2. Unlike the stimulatory effect of RCAN1-WT, the RCAN1^1–145^ fragment failed to increase the interaction between IRAK1 and TRAF6 (Figure 5C). These results indicate that LRRK2 stimulates inflammatory signaling through potentiation of RCAN1-mediated positive modulation of IL-1β signaling.

### LRRK2 Enhances the Auto-Phosphorylation of TAK1

**Figure 6:**
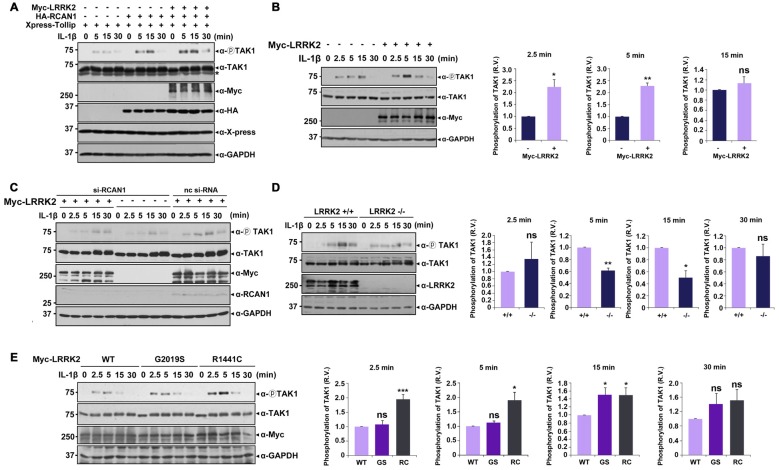
**LRRK2 potentiates TAK1 auto-phosphorylation in response to IL-1β stimulation. (A)** HEK293/IL-1RI cells were transfected for 24 h with Xpress-Tollip, HA-RCAN1, or Myc-LRRK2 alone or in combination and treated with 10 ng/ml IL-1β for the indicated times. Cell lysates were immunoblotted with anti-phospho-TAK1 (Thr187), anti-TAK1, or anti-LRRK2 antibodies. GAPDH served as a loading control. Asterisks indicate non-specific proteins.** (B)** HEK293/IL-1RI cells were mock-transfected or transfected for 24 h with Myc-LRRK2 and treated with 10 ng/ml IL-1β for the indicated times. Cell lysates were immunoblotted with anti-phospho-TAK1 (Thr187), anti-TAK1, or anti-LRRK2 antibodies. GAPDH served as a loading control (left panel). Quantitation of the images was presented (right panel). Error bars indicate ± SEM in triplicate experiments (*p < 0.05; **p < 0.01).** (C)** Where specified, HEK293/IL-1RI cells were mock-transfected or transfected for 24 h with nonspecific control si-RNA (nc-si-RNA; 200 nM), *RCAN1*-siRNA (si-RCAN1; 200 nM), or Myc-LRRK2-WT alone or in combination. Cells were then treated with 10 ng/ml IL-1β for the indicated times. RCAN1 knockdown was verified using western blotting with an anti-RCAN1 antibody. Cell lysates were immunoblotted with anti-phospho-TAK1 (Thr187), anti-TAK1, or anti-Myc antibodies. GAPDH served as a loading control. **(D)** *LRRK2* +/+ or *LRRK2* −/− MEFs were treated with 50 ng/ml IL-1β for the indicated times. Cell lysates were immunoblotted with anti-phospho-TAK1 (Thr187), anti-TAK1, or anti-LRRK2 antibodies. GAPDH served as a loading control (left panel). The intensity of phospho-TAK1 bands was quantified by densitometry using Multi Gauge v3.1 software (right panel; *n* = 3 in **D** and *n* = 4 in **E**). Individual band intensities were normalized to that for total TAK1 for each experimental group. Error bars indicate ± SEM in triplicate experiments (*p < 0.05, **p < 0.01 in **D**; and *p < 0.05, ***p < 0.001 in **E**).** (E)** HEK293/IL-1RI cells were transfected for 24 h with plasmids encoding Myc-tagged LRRK2-WT or the LRRK2-G2019S and LRRK2-R1441C mutants, and then treated with 10 ng/ml IL-1β for the indicated times. Cell lysates were immunoblotted with anti-phospho-TAK1 (Thr187), anti-TAK1, or anti-Myc antibodies. GAPDH served as a loading control.

Upon stimulation with IL-1β, K63-linked polyubiquitination of TRAF6 occurs after IRAK1-TRAF6 complex formation, which subsequently activates TAK1 (Sun et al., 2004). Based on reports that TAK1 auto-phosphorylation at Thr187 is closely related to its kinase activity (Kajino et al., 2006; Yu et al., 2008), we next investigated the effects of LRRK2 on basal and IL-1β-induced TAK1 activity. After transfection with Xpress-Tollip, HA-RCAN1, or Myc-LRRK2 alone or in combination, HEK293/IL-1RI cells were left untreated or stimulated with IL-1β. Immunoblot analyses of cell lysates with anti-phospho-TAK1 antibodies confirmed phosphorylation of TAK1 at T187 in response to IL-1β stimulation; there was no significant activation of TAK1 in the absence of IL-1β. Treatment with IL-1β led to a significant induction of TAK1 phosphorylation, which reached a maximum level after 15 min. When cells were co-transfected with Tollip and RCAN1, TAK1 activity was more elevated compared with when cells were transfected with Tollip alone. Furthermore, LRRK2 over-expression increased TAK1 activity under resting conditions and after IL-1β stimulation (Figure 6A). Interestingly, LRRK2 overexpression caused much stronger TAK1 phosphorylation, reaching its maximum at 5 min after IL-1β stimulation (Figure 6B). However, this effect was greatly diminished by RCAN1 knockdown (Figure 6C). These data suggest that RCAN1 could be a key regulator of LRRK2-induced IL-1β signaling.

We further examined the effect of *LRRK2* knockdown on TAK1 activity using *LRRK2* −/− and *LRRK2* +/+ MEFs. Immunoblot analyses of cell lysates with a phospho-TAK1 antibody showed that TAK1 activity was significantly decreased in *LRRK2* −/− MEF compared with *LRRK2* +/+ MEF (Figure 6D). When we investigated the effects of two PD-associated LRRK2 mutants (LRRK2-G2019S and LRRK2-R1441C) on TAK1 activity, both mutants enhanced TAK1 activity (Figure 6E), consistent with other results reported herein. Collectively, these data suggest that LRRK2 potentiates TAK1 activity in response to treatment with IL-1β, which consequently stimulates IL-1β mediated downstream signaling.

### LRRK2 Enhances Production of IL-8 and Activation of NF-κB in Response to IL-1β

**Figure 7:**
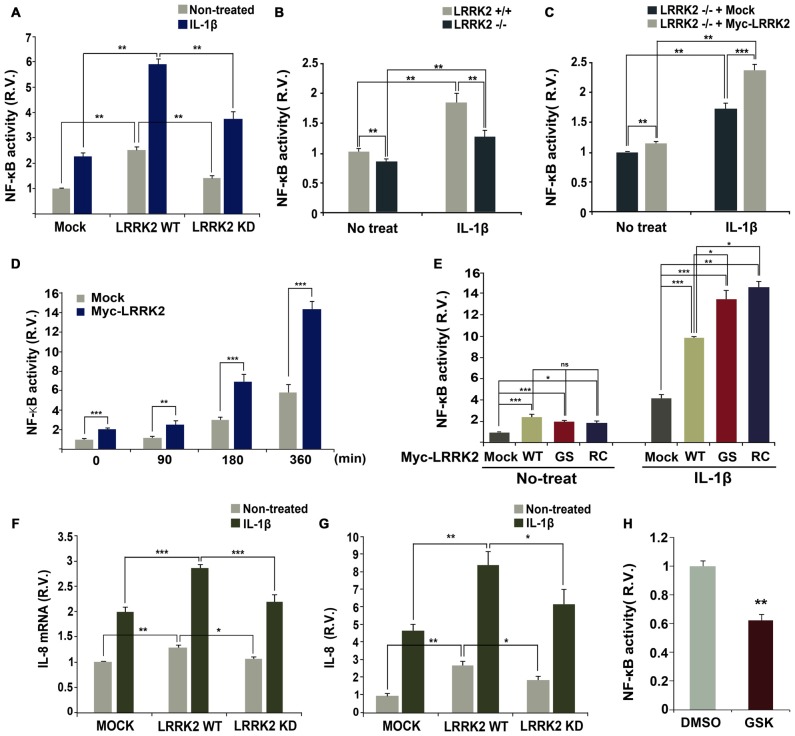
**LRRK2 enhances NF-κB activation and production in response to IL-1β. (A)** HEK293/IL-1RI cells were transfected with NF-κB-responsive luciferase reporter and a control Renilla luciferase reporter alone or in combination with Myc-LRRK2-WT or Myc-LRRK2-D1994A (LRRK2-KD). Cells were then treated for 6 h with 10 ng/ml IL-1β. Relative luciferase activity was measured and normalized to Renilla activity. Error bars indicate ± SEM in triplicate experiments (**A,B**; **p < 0.01). **(B,C)** Where specified, *LRRK2* +/+ and *LRRK2* −/− MEFs were transfected with NF-κB-responsive luciferase reporter and a control Renilla luciferase reporter **(B)**. Or else, *LRRK2* −/− MEF cells were transfected with NF-κB-responsive luciferase reporter and a control Renilla luciferase reporter alone or in combination with Myc-LRRK2-WT **(C)**. Cells were then treated for 6 h with 50 ng/ml IL-1β. The relative luciferase activity was measured and normalized to Renilla activity. **(D,E)** HEK293/IL-1RI cells were transfected with NF-κB-responsive luciferase reporter and a control Renilla luciferase reporter in combination with empty vector or Myc-LRRK2-WT, or its LRRK2-G2019S or LRRK2-R1441C mutants. Cells were then treated for the indicated times **(D)** or 6 h **(E)** with 10 ng/ml IL-1β. Their relative luciferase activities were measured and normalized to Renilla activity (**D,E**; *p < 0.05; **p < 0.01; ***p < 0.001). **(F,G)** HEK293 cells were either mock-transfected or transfected with Myc-LRRK2-WT or Myc-LRRK2-KD. Cells were then left untreated or stimulated for 8 **(F)** or 24 h **(G)** with 25 ng/ml IL-1β. Real-time PCR analysis of IL-8 mRNA was performed using IL-8 primers (**F**; *p < 0.05; **p < 0.01; ***p < 0.001). Otherwise, the amount of secreted IL-8 protein was measured by enzyme-linked immunosorbent assay (ELISA) kit (**G**; *p < 0.05; **p < 0.01). Results are representative of six and five independent experiments. **(H)** RAW264.7 cells were transfected with NF-κB-responsive luciferase reporter and a control Renilla luciferase reporter, and treated for additional 5 h with 5 μM GSK2578215A (GSK). The relative luciferase activity was measured and normalized to Renilla activity. Error bars indicate ± SEM in triplicate experiments (**p < 0.01).

Stimulation with IL-1β elicits transcriptional activation of NF-κB in target cells (Yamazaki et al., 2009; Fan et al., 2010). To investigate potential effects of LRRK2 on NF-κB activity, a luciferase NF-κB reporter gene assay was performed in the absence or presence of LRRK2. Over-expression of wild-type LRRK2 dramatically enhanced NF-κB activity in response to IL-1β treatment, but this effect was not observed with LRRK2-KD (Figure 7A). To further confirm the stimulatory effect of LRRK2 on NF-κB activity, we compared the activity of NF-κB in *LRRK2* −/− and *LRRK2* +/+ MEFs. NF-κB activity in *LRRK2* −/− MEFs was reduced compared with *LRRK2* +/+ MEFs in response to IL-1β treatment (Figure 7B). In addition, the reduced NF-κB activity was rescued back to the control level by reintroduction of LRRK2-WT into* LRRK2* −/− MEFs (Figure 7C), confirming a positive regulatory effect of LRRK2 on NF-κB activity. We also examined the time-dependence of NF-κB activity with IL-1β treatment. HEK293/IL-1RI cells were mock-transfected or transfected with LRRK2-WT. NF-κB reporter dual luciferase assays showed that NF-κB activity more rapidly increased in the presence of LRRK2 (Figure 7D). We additionally assessed the effects of PD-associated pathogenic LRRK2 mutants (LRRK2-G2019S and LRRK2-R1441C) on NF-κB activity. Consistent with our previous findings that LRRK2 stimulates IL-1β-mediated downstream signaling, the two PD-associated mutants, having enhanced kinase activities, stimulated NF-κB activity more than did their wild-type counterpart (Figure 7E).

Lastly, we investigated whether the positive effect of LRRK2 on IL-1R signaling events leads to the production and subsequent secretion of inflammatory cytokines, such as IL-8, in HEK293/IL-1RI cells in response to stimulation with IL-1β. Consistent with the stimulatory effect of LRRK2 on the upstream components of IL-1R signaling, the presence of wild-type LRRK2 increased levels of IL-8 mRNA, as determined by real-time PCR (Figure 7F). Additionally, wild-type LRRK2 stimulated secretion of IL-8, as determined by ELISA (Figure 7G). Interestingly, when compared with mock-transfected controls, cells transfected with LRRK2-WT displayed enhanced NF-κB activity in the absence of IL-1β treatment, which greatly increased with IL-1β treatment (Figures 7A,E). However, this effect was not observed in cells transfected with LRRK2-KD (Figure 7A). Similarly, the synthesis of IL-8 mRNA and protein greatly increased in cells transfected with LRRK2-WT alone, but not with LRRK-KD (Figures 7F,G). We next examined whether LRRK2 is necessary for enhanced NF-κB activity in immune cells as well as in HEK293/IL-1RI cells and MEFs. Pretreatment of RAW264.7 cells with LRRK2 inhibitor caused a decrease in NF-κB activity (Figure 7H). Collectively, these results suggest that LRRK2 acts as a positive regulator of IL-1β-mediated signaling, leading to up-regulation of inflammatory cytokines in response to stimulation with IL-1β.

## Discussion

The present study demonstrated that PD-associated LRRK2 positively modulates IL-1β-mediated inflammatory signaling through selective phosphorylation of RCAN1. Accordingly, excessive brain inflammation is increasingly recognized as a major factor participating in brain diseases, including Alzheimer’s disease (AD) and PD. Numerous reports revealed that inflammatory process by immune cell elicits neuronal and non-neuronal cell death in brain (Broughton et al., 2013). Not only that, some molecules closely associated with inflammatory processes, such as serum homocysteine, uric acid, plasma cystatin C, and high-density lipoprotein are commonly regarded as the biomarkers of multiple system atrophy, AD, and vascular dementia (Chen et al., 2015; Wang et al., 2017). Furthermore, inflammation seems to be important for the pathogenesis of PD. For example, neurotoxin MPP^+^ treatment, which causes the dopaminergic neuron loss and PD-like symptoms, promotes the production and release of various cytokines through TLR4 signaling in BV2 cells (Zhou et al., 2016). In addition, many reports suggest that specific molecules and their receptors are closely related to and regulate the progression of PD. For example, Nur77, an important regulator of neuroinflammation and dopaminergic neurodegeneration, seems to play a role in 6-hydroxydopamine (OHDA)-induced model of PD (Gao et al., 2016). Treatment of 6-OHDA changed intracellular localization of Nur77 from nucleus to cytosol, triggering its transcriptional activation and subsequent cell death via aggravating mitochondrial impairment and endoplasmic reticulum stress (Gao et al., 2016). Or else, memantine, an NMDA receptor antagonist and commonly used AD medication, suppresses the expression of Nur77 and Nurr1 through anti-inflammation and anti-mitochondrial impairment (Wei et al., 2016). The nuclear translocation of Nur77 was also reduced by memantine (Gao et al., 2016; Wei et al., 2016). Anti-inflammatory agent, simvastatin, also affects dopaminergic neuronal degeneration. For example, simvastatin suppressed 6-OHDA-induced neurotoxicity and increased the levels of IL-6, TNF-α and COX_2_ (Xu et al., 2013). Moreover, the functional and pathological alteration of adrenergic receptors, acetylcholine receptors, and dopamine receptors contribute cognitive deficits in PD patients, underlying the involvement of variety signaling pathways (Xu et al., 2012). All these reports including the current study further validate the hypothesis that inflammation is an important factor for PD.

Several studies have recently reported a mechanistic link between LRRK2 and innate immunity and propose a functional role for LRRK2 in the positive regulation of NF-κB-dependent gene transcription (Kim et al., 2007; Gardet et al., 2010). Despite intensive investigation, the underlying mechanism of LRRK2-mediated inflammatory response and the signaling pathways involved have not yet been clearly elucidated. Two putative upstream regulators of LRRK2 have been identified: ERK5 and IKK. Activation of LRRK2 can be induced through ERK5 signaling by treatment with interferon-γ (Kuss et al., 2014). Additionally, exposure to LPS increases levels of phosphorylated LRRK2, whereas treatment with IKK inhibitors inhibits LRRK2 phosphorylation (Dzamko et al., 2012). While these two proteins appear to act upstream of LRRK2, there is not much known about downstream substrates of LRRK2 during IL-1R/TLR signaling. Here, we present several lines of evidence demonstrating that RCAN1 positively regulates in IL-1β signaling pathway, which is potentiated by LRRK2-mediated phosphorylation of RCAN1. We show that LRRK2 physically interacts with RCAN1 and that the N-terminal 30–95 amino acid region of RCAN1 is critical for their interaction. LRRK2 also directly phosphorylates RCAN1, which is greatly increased with stimulation by IL-1β.

Like others proteins, RCAN1 is modulated by various post-translational modifications. Among them, phosphorylation is the most prominent regulatory mechanism. A number of protein kinases regulate the functional activity and/or stability of RCAN1. For example, protein kinase A and NF-κB-inducing kinase phosphorylate RCAN1 and increase its half-life, which consequently enhances the inhibitory function of RCAN1 in NFAT signaling (Lee et al., 2008; Kim et al., 2012). In addition, Dyrk1A also promotes the inhibitory effect of RCAN1 on calcineurin through its kinase activity (Jung et al., 2011). In contrast, MEK5-BMK1 signaling and TAK1 suppress RCAN1 function, increasing NFAT activity (Abbasi et al., 2006; Liu et al., 2009). Furthermore, the yeast protein Mck1, a member of the GSK-3 family of protein kinases, phosphorylates Rcn1, enhancing downstream calcineurin signaling (Hilioti et al., 2004). Here, we identified LRRK2 as a novel kinase acting on RCAN1. Unlike other kinases, LRRK2 has no effect on RCAN1 protein stability. While we could not determine the exact sites of RCAN1 targeted by LRRK2, it may modify other sites distinct from those of other kinases. Further experimentation is required to investigate this hypothesis.

The protein binding partners of RCAN1 are classified into two groups, depending on their involvement in the calcineurin signaling pathway. For example, RCAN1 binds to the cAMP response element-binding protein (CREB) and positively regulates the CREB pathway via modulation of calcineurin activity (Kim and Seo, 2011). Under oxidative stress conditions, RCAN1 plays a protective role through regulation of the CREB pathway (Kim et al., 2013). Furthermore, RCAN1 negatively regulates vesicle recycling and endocytosis via inhibition of calcineurin activity (Zanin et al., 2013). As example of a binding partner not associated with calcineurin activity is the interaction between RCAN1 and phospho-FMRP, which induces spine morphogenesis and local protein synthesis in dendritic spines (Wang et al., 2012). We previously showed that the binding of RCAN1 to Tollip, an inhibitor of IL-1β signaling, potentiates IL-1β-mediated transduction (Lee et al., 2009), which also occurs through a calcineurin-independent pathway. The present study reveals that LRRK2 falls under the second group of RCAN1-binding proteins. Moreover, LRRK2 does not affect RCAN1 binding to calcineurin, nor downstream NFAT activity (Han and Chung, unpublished observation). These results suggest that LRRK2 modulates RCAN1 independently from downstream calcineurin-signaling pathway.

In the present work, we demonstrate that LRRK2 increase binding of RCAN1 to Tollip. LRRK2 stimulated the downstream formation of a signalosome complex involving IRAK1 and TRAF6, and facilitated TAK1 auto-phosphorylation in response to stimulation by IL-1β. Consistent with other present findings, LRRK2 dramatically increased NF-κB activity in response to IL-1β treatment. Furthermore, LRRK2 potentiated the production of IL-8 and its extracellular release via NF-κB activation in response to IL-1β treatment. According to the previous report, myeloid cell recruitment was reduced in LRRK2-knockout rat by LPS treatment, leading to the suppression of dopaminergic neurodegeneration (Daher et al., 2014). The present work also suggests that chemokine IL-8 release could play a role in LRRK2-mediated toxicity by recruiting the myeloid cells to midbrain region. In contrast to our present findings, previous studies reported that the kinase-dead LRRK2 mutant and its wild-type counterpart both promote NF-κB activity (Kim et al., 2007; Gardet et al., 2010). This discrepancy may be due to the different stimuli and cell types used. Therefore, other immune cell types should be tested. However, our findings were corroborated by data demonstrating that two PD-associated LRRK2 mutants, both possessing enhanced kinase activity, correspondingly increased phosphorylation of RCAN1 compared with wild-type LRRK2. These results also provide additional insight into the role of these pathogenic LRRK2 mutations during putative RCAN1-mediated IL-1-induced inflammation in PD. Our results may serve as a basis for future work in RCAN1 knockout/transgenic models and PD-associated models over-expressing these LRRK2 mutants.

Inflammation and the inflammatory response are important contributing factors to the pathogenesis of various neurodegenerative diseases, including PD. Collectively, the present work suggests that alteration of LRRK2 function might play a significant role in the development of these conditions.

## Author Contributions

KAH and KCC formulated the hypothesis, initiated and organized the study and wrote the manuscript. KAH, JWU, HK, WS and KCC analyzed data. KAH, JYS, LY and SAC performed experiments.

## Funding

This work was supported by grants from the National Research Foundation of Korea (NRF) funded by the Ministry of Science, ICT and Future Planning (2014M3C7A1064545 and 2015R1A2A2A01003080 to KCC), South Korea. This work was also supported in part by the Yonsei University Future-leading Research Initiative of 2015 (2015-22-0055 to KCC).

## Conflict of Interest Statement

The authors declare that the research was conducted in the absence of any commercial or financial relationships that could be construed as a potential conflict of interest.
